# Expression of Antioxidant Enzymes in Patients with Uterine Polyp, Myoma, Hyperplasia, and Adenocarcinoma

**DOI:** 10.3390/antiox8040097

**Published:** 2019-04-11

**Authors:** Ana Todorović, Snežana Pejić, Ljubica Gavrilović, Ivan Pavlović, Vesna Stojiljković, Nataša Popović, Snežana B. Pajović

**Affiliations:** Laboratory of Molecular Biology and Endocrinology, “Vinča” Institute of Nuclear Sciences, University of Belgrade, P.O. Box 522, 11001 Belgrade, Serbia; snezana@vin.bg.ac.rs (S.P.); gljubica@vin.bg.ac.rs (L.G.); pavlovic@vin.bg.ac.rs (I.P.); vesnas@vin.bg.ac.rs (V.S.); natasap@vin.bg.ac.rs (N.P.); pajovic@vin.bg.ac.rs (S.B.P.)

**Keywords:** antioxidant enzymes, polyps, myoma, hyperplasia, adenocarcinoma

## Abstract

We previously found that compared to patients with benign uterine diseases (polyps, myomas), patients with premalignant (hyperplasia simplex and complex) and malignant (adenocarcinoma) lesions had enhanced lipid peroxidation and altered uterine antioxidant enzyme (AOE) activities. To further elucidate the mechanism of the observed changes, we examined protein and mRNA levels of copper-zinc superoxide dismutase (CuZnSOD), catalase (CAT), glutathione peroxidase (GPx), glutathione reductase (GR), and transcription factor Nrf2. We also examined correlations of AOE expression with AOE activity, lipid hydroperoxides (LOOH) level, and level of Nrf2. Our results showed decreased CuZnSOD, CAT, and Nrf2 levels, and increased GPx and GR levels in hyperplasias, while in patients with adenocarcinoma, the level of CAT was decreased and GR was increased, compared to benign groups. Similar changes in mRNA levels were also detected, indicating predominantly translational control of the AOE expression. The positive correlation of enzyme expression/activity was recorded for CuZnSOD, GPx, and GR, but only among groups with benign diseases. Only GR and GPx expressions were positively correlated with LOOH. Nrf2 protein was positively correlated with mRNA levels of CuZnSOD and GR. Observed results indicate involvement of diverse redox mechanisms in etiopathogenesis of different gynecological diseases, and may improve redox-based approaches in current clinical practice.

## 1. Introduction

During the life cycle, aerobic organisms are exposed to a number of endogenous and exogenous sources of reactive oxygen species (ROS). To achieve redox homeostasis they developed a powerful antioxidant system (AOS) [1], whose main enzyme components are superoxide dismutase (SOD), catalase (CAT), glutathione peroxidase (GPx), and glutathione reductase (GR). The transcription factor Nrf2 is responsible for regulating a battery of antioxidant and detoxification enzymes [2,3], as well as processes such as stress response, proliferation, and proteasomal degradation [4,5]. Nrf2 was also identified as an important transcription factor regulating development, progression, and chemoresistance of cancer [6]. 

Cancer develops over three stages, initiation, promotion, and progression, and oxidative stress is associated with each of them [7]. Endometrial cancer ranks as the fourth most common neoplasm in women from European countries [8]. It has been shown that premalignant changes precede the malignant transformation of the uterus, which is why hyperplasia may be considered as a precursor of endometrial cancer [9,10,11]. Despite numerous studies, the molecular processes involved in multi-stage development of endometrial cancer are still not completely known.

We have previously shown that gonadotropins influence the antioxidant enzyme (AOE) activity in women with uterine hyperplasia simplex, hyperplasia complex, leiomyoma, and polyps [12,13,14,15]. Also, the extent of AOE and lipid hydroperoxides (LOOH) alterations varied with the examined gynecological diagnosis, including adenocarcinoma [16]. The aim of this study was to further clarify the mechanism responsible for the observed AO alterations. Therefore, we examined the changes of protein and mRNA levels of copper-zinc superoxide dismutase (CuZnSOD), catalase (CAT), glutathione peroxidase (GPx), glutathione reductase (GR), and Nrf2 in uterine tissue of patients with benign (polyps and myomas), premalignant (hyperplasia simplex and hyperplasia complex), and malignant (adenocarcinoma) transformations. We also examined correlations of AOE expression with the AOE activity, lipid hydroperoxides (LOOH) level, and level of Nrf2. Results showed significant differences in AO parameters among examined groups of gynecological patients, and indicated their important role in pathophysiological processes in endometrium. 

## 2. Materials and Methods 

### 2.1. Subjects

A total of 79 subjects from the Department of Gynecology and Obstetrics admitted for routine checkups or for abnormal uterine bleeding were included in this prospective study. According to diagnosis and histological results, subjects were divided into five groups: polypus endometrii (PE, *n* = 16, median age 43 ± 2 yr, range 31–57 yr), uterus myomatosus (UM, *n* = 12, median age 46 ± 2 yr, range 34–54 yr), hyperplasia simplex endometrii (SH, *n* = 25, median age 49 ± 3yr, range 32–60 yr), hyperplasia complex endometrii (CH, *n* = 21, median age 47 ± 3 yr, range 45–59 yr), and adenocarcinoma endometrii, stage I (ACE, *n* = 5, median age 58 ± 4yr, range 49–61 yr). Patients’ characteristics are summarized in Table 1. The research was approved by the Ethics Committee of the Clinical Center (No. 27/06-2006), and in accordance with the World Medical Association Declaration of Helsinki. Samples were taken after provision of informed consent. The patients received no medical treatment in the 6 months before sampling.

### 2.2. Samples and Methods

Tissue samples were removed and homogenized on ice in a phosphate buffer of pH 7.8 (1:2 g/mL). Enzyme assays and concentration of LOOH were measured according to the procedures described previously [13]. Protein concentration in prepared samples was measured by Lowry et al.’s method [17], and used to express enzyme activities of CuZnSOD, CAT, GPx, and GR as Units (U) or mU per milligram of protein (U or mU/mg protein). Level of LOOH was also expressed relative to protein

Western blot was performed as previously described [18]. Immuno-blot was conducted using antibodies against CuZnSOD (Stressgen, 1:7500), GPx1 (Santa Cruz 1:500), GR (Santa Cruz, 1:500), actin (Abcam, 1:500), Nrf2 (Santa Cruz, 1:500), and horseradish peroxidase (HRP) conjugated secondary antibodies. Immunoreactive proteins were visualized using enhanced chemiluminescence (ECL) detection. Densitometry of protein bands, normalized to β-actin, was performed by Image J software. 

Quantitative RT-PCR analysis: Total RNA was isolated from endometrial tissue using Trizol reagent (Invitrogen, USA) according to the manufacturer’s protocol. RNA purity was evaluated by determining the 260/280 ratio (GeneQuant Pro, Biochrom, UK), and 1 μg of total RNA was used for cDNA synthesis with a High Capacity RNA-to-cDNA Master Mix (Applied Biosistems, USA), as described in the manufacturer’s specifications. TaqManPCR assays were performed using Assay-on-Demand Gene Expression Products (Applied Biosystems, USA) for CuZnSOD (Hs00172187_m1), CAT (Hs00156308_m1), GPx (Hs02516751_s1), GR (Hs00167317_m1), Nrf2 (Hs00232352_m1), and POLR2A (Hs00172187_m1). RT-qPCR assay was performed with ABI Prism 7000 Sequence Detection System (Applied Biosystems, Foster City, CA, USA) under the following conditions: 50 °C (2 min), 95 °C (10 min), followed by 40 cycles of 95 °C (15 s), 60 °C (1 min). All experiments were performed in triplicate, and the data were normalized to POLR2A. 

### 2.3. Statistical Analysis

Statistical analysis was performed using the Kruskal-Wallis method followed by Dunn’s multiple-comparison test. Spearman’s rank correlation coefficient was used to investigate associations between enzyme protein levels and activities, as well as between enzyme protein levels and lipid peroxidation. A 2-tailed p < 0.05 was considered statistically significant. All data were analyzed using GraphPad Prism software (version 4.0, GraphPad Software, Inc., San Diego, CA, USA).

## 3. Results

**Figure 1:**
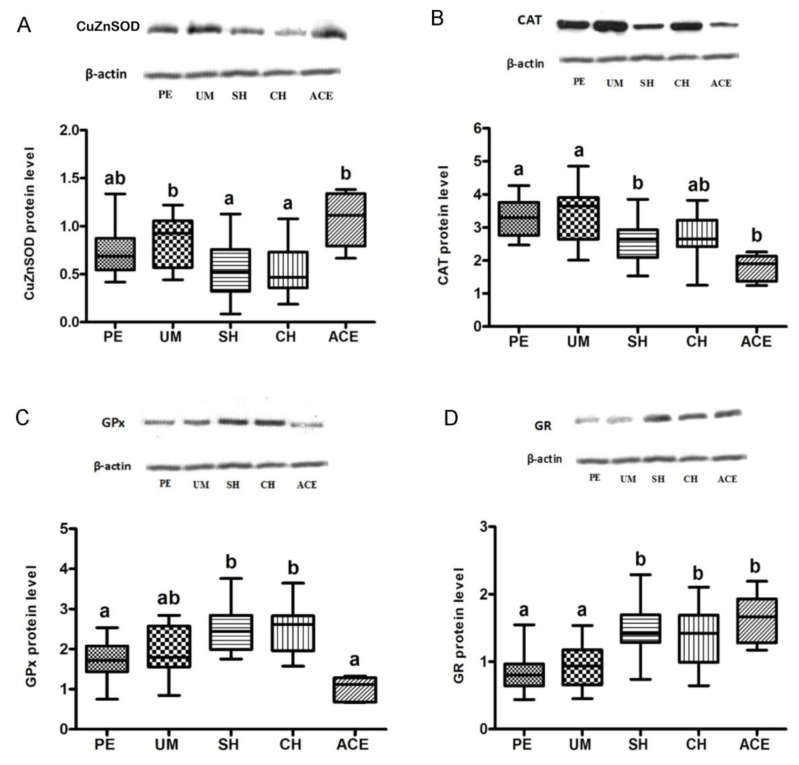
Representative Western blot bands and quantitative analysis of CuZnSOD (**A**), CAT (**B**), GPx (**C**), and GR (**D**) in endometrium of patients diagnosed with polypus endometrii (PE), uterus myomatosus (UM), simple hyperplasia (SH), complex hyperplasia (CH), and adenocarcinoma endometrii (ACE). Mean protein levels (± SD) are represented by the box; medians are plotted inside a box; the whiskers extend to the 5th and 95th percentiles. Values with the same letter (a, b) did not differ significantly from each other (*p* > 0.05).

Endometrial protein level of CuZnSOD in patients with myoma was comparable to that observed in patients with polyps and adenocarcinoma, whereas in patients with simple or complex hyperplasia it was significantly lower (*p* = 0.035, *p* = 0.046, respectively; Figure 1A). 

There was no significant difference in CAT level between the patients with polyps and myoma, or compared to those with hyperplasia complex. In comparison with polyps and myoma, the protein level of CAT was decreased in subjects with hyperplasia simplex (*p* = 0.005, *p* = 0.045, respectively; Figure 1B) and adenocarcinoma (*p* = 0.001, *p* = 0.002, respectively; Figure 1B).

Patients with polyps and myoma had a comparable GPx level to that observed in patients with adenocarcinoma. Compared to women with polyps and adenocarcinoma, the GPx level was significantly higher in patients with hyperplasia simplex (*p* = 0.001, *p* < 0.001, respectively) and hyperplasia complex (*p* = 0.001, *p* < 0.001, respectively; Figure 1C).

Compared to patients with polyps and myoma, significant elevation of GR level was recorded in groups with hyperplasia simplex (*p* < 0.001, *p* = 0.005, respectively), hyperplasia complex (*p* = 0.001, *p* = 0.049, respectively), and adenocarcinoma (*p* = 0.007, *p* = 0.050, respectively) (Figure 1D).

**Figure 2:**
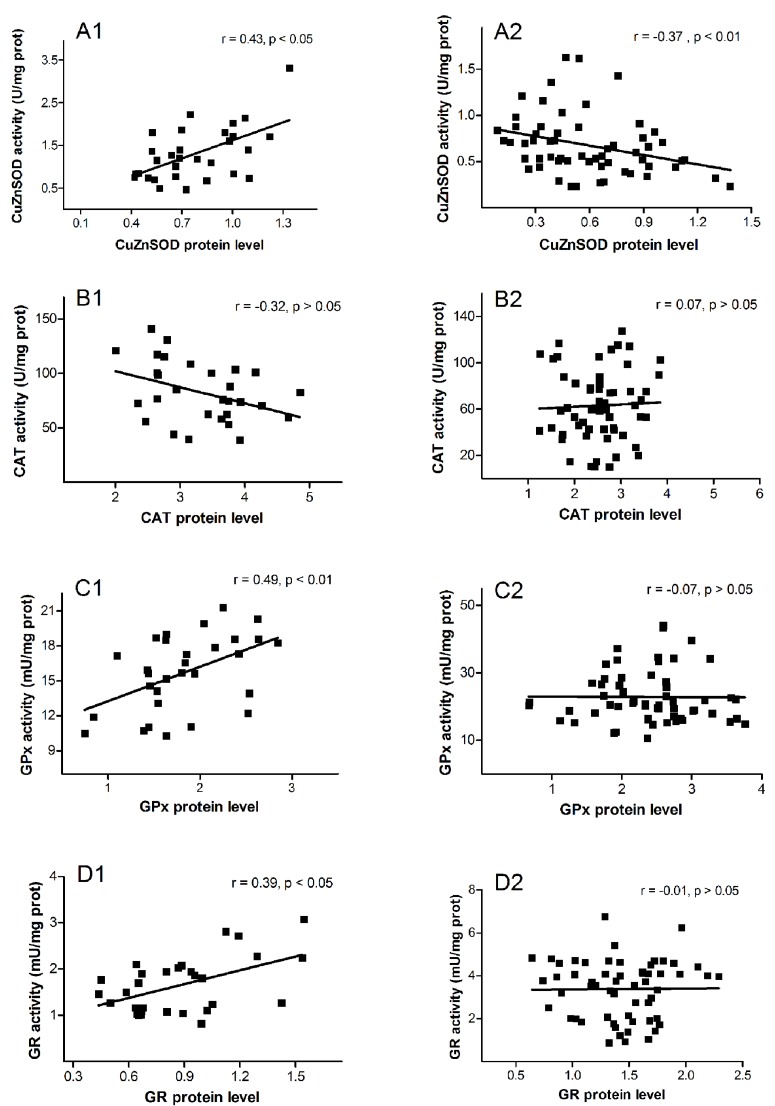
Scatter plots of the activity against quantity of CuZnSOD: (**A1**) benign and (**A2**) premalignant and malignant groups. CAT: (**B1**) benign and (**B2**) premalignant and malignant groups. GPx: (**C1**) benign and (**C2**) premalignant and malignant groups. GR: (**D1**) benign and (**D2**) premalignant and malignant groups. Benign group was composed of polyps and myomas groups. Premalignant/malignant group consisted of hyperplasia simplex, hyperplasia complex, and adenocarcinoma. Enzyme activities were expressed as Units (U) or mU per milligram of total cell protein (U or mU/mg protein). Protein levels were normalized to β-actin.

Correlation analysis of AOE activity against their protein levels for benign and premalignant/malignant groups of patients is shown in Figure 2. The positive correlation was observed in benign group for CuZnSOD (*r* = 0.43, *p* = 0.019; Figure 2A1), GPx (*r* = 0.49, *p* = 0.006; Figure 2C1), and GR (*r* = 0.39, *p* = 0.033; Figure 2D1). There was no activity/level correlation in premalignant/malignant group, except for CuZnSOD where negative activity/level correlation was recorded (*r* = −0.37, *p* = 0.005; Figure 2A2).

**Figure 3:**
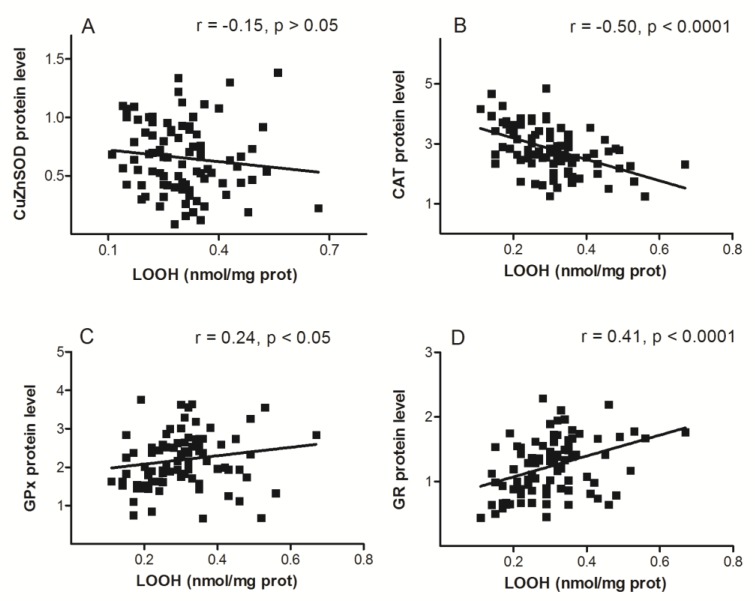
Scatter plots of lipid hydroperoxides—LOOH level against proteins levels of copper-zinc superoxide dismutase—CuZnSOD (**A**), catalase—CAT (**B**), glutathione peroxidase—GPx (**C**), and glutathione reductase—GR (**D**). Protein levels were normalized to β-actin.

LOOH level was negatively correlated to protein level of CAT (*r* = −0.50, *p* < 0.001; Figure 3B), and positively correlated with levels of GPx (*r* = 0.24, *p* = 0.026; Figure 3C) and GR (*r* = 0.41, *p* < 0.001; Figure 3D), whereas no correlation was found with the level of CuZnSOD.

**Figure 4:**
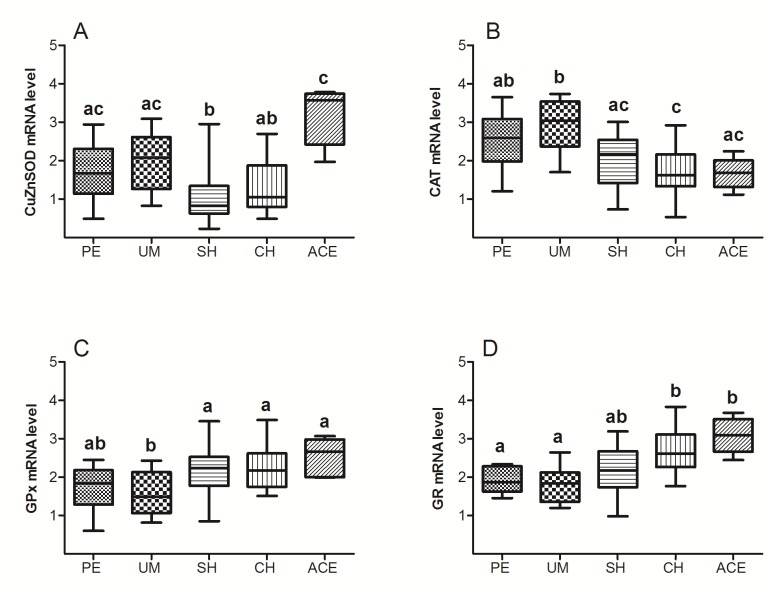
Box plots represent relative gene expression for CuZnSOD (**A**), CAT (**B**), GPx (**C**), and GR (**D**) in endometrium of patients diagnosed with polypus endometrii (PE), uterus myomatosus (UM), simple hyperplasia (SH), complex hyperplasia (CH), and adenocarcinoma endometrii (ACE). Data were determined by qRT PCR and normalized to endogenous POLR2A expression. Mean mRNA levels (± SD) are represented by the box; medians are plotted inside a box; the whiskers extend to the 5th and 95th percentiles. Values with the same letter (a, b) did not differ significantly from each other (*p* > 0.05).

Data on Figure 4A indicated that compared to the groups with polyps and myoma, CuZnSOD mRNA level was lowered in group with SH and it was not significantly changed in adenocarcinoma group. In patients with hyperplasias and adenocarcinoma, the CAT mRNA level showed a tendency to decrease, while the GPx and GR mRNAs inclined toward increase, compared to PE and UM groups (Figure 4B–D).

**Figure 5:**
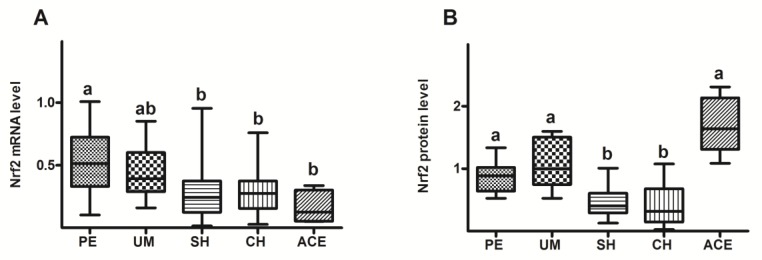
Box plots represent relative levels of Nrf2 mRNA (**A**) and Nrf2 protein (**B**) in endometrium of patients diagnosed with polypus endometrii (PE), uterus myomatosus (UM), simple hyperplasia (SH), complex hyperplasia (CH), and adenocarcinoma endometrii (ACE). Mean mRNA and protein levels (± SD) are represented by the box; medians are plotted inside a box; the whiskers extend to the 5th and 95th percentiles. Values with the same letter (a, b) did not differ significantly from each other (*p* > 0.05).

Compared to the groups with polyps and myoma, Nrf2 mRNA level was lowered in the groups with hyperplasias and adenocarcinoma (Figure 5A). Data on Figure 5B showed that Nrf2 protein level was significantly decreased in patients with hyperplasia (SH and CH), related to the groups with polyps, myoma, and adenocarcinoma.

Nrf2 protein level was positively correlated to mRNA levels of CuZnSOD and GR, negatively correlated to mRNA level of GPx, and not correlated to the mRNA level of CAT.

## 4. Discussion

In this study, we recorded a different expression of AOE in transformed endometrial tissue. Furthermore, we noticed that positive correlation of AOE expression/activity observed in patients with benign endometrial changes was mostly lost in premalignant and malignant groups, pointing to different molecular mechanisms that regulate AOE levels in examined gynecological diseases.

In patients with hyperplasia simplex and hyperplasia complex, the CuZnSOD expression was decreased compared to myoma group (Figure 1A), suggesting that reduced CuZnSOD levels favor the formation of premalignant lesions. Reduced levels of CuZnSOD were also found during the malignant transformation of lung [19], prostatic [20], and renal cells [21]. According to findings of Oberley and Oberley [22] on papillomas and squamous cell carcinoma, the SOD and CAT reduction leads to a pro-oxidant state of cells, facilitating tumorigenesis. Similarly, Bostwick et al. [23] and Srivastava et al. [24] observed that SOD level decreased during the progression of cervical and prostate cancer. Our results indicate that in adenocarcinoma tissue, the CuZnSOD protein level was increased compared to hyperplasia groups (Figure 1A), although the same increase was not observed previously regarding CuZnSOD activity [11]. This may suggest that ROS present in tumor cells stimulate the expression of CuZnSOD protein, but simultaneously partly reduce CuZnSOD activity, as already noted in colorectal cancer tissue [25]. 

Regulation of CuZnSOD expression seems to be performed at the transcriptional level, since the trend of changes in CuZnSOD mRNA (Figure 4A) was followed by those of CuZnSOD protein. Such regulation can be provided by several transcriptional factors [26], most probably by redox-dependent regulators, such as Egr1 (Early Growth Response-1), AP1 (Activating Protein 1), NF-κB (Nuclear Factor-KappaB), and Nrf2 [27]. Altered level of O_2_^−^ can further affect downstream cell signaling targets and eventually affect cell sensitivity, proliferation, and even death. It has been shown that in hepatic stellate cells, superoxide anion can induce apoptosis through the mechanism that involves reducing cellular glutathione level and activating Jun N-terminal kinase (JNK) and p38 [28], or by inducing death receptor CD95 (Fas) on the cell surface [29]. Also, research on the M14 melanoma cells indicated that intracellular O_2_^−^ regulates tumor cell sensitivity through the caspase activation pathway [30], while others showed that O_2_^−^ might function as a mitogenic stimuli by inducing the expression of early growth-related genes, such as c-fos and c-jun [31].

Observed changes in CuZnSOD level during the course of malignant transformation may have clinical significance due to the influence that cell redox status has on tumor progression. The study of Skrzycki et al. [25] suggested that changes in protein level and activity of SOD isoenzymes might be an adaptive response to the oxidative stress. As SOD isoenzymes are one of the main redox state controllers, therapies affecting SOD activity or expression at different stages of cancer could aid in elimination of transformed cells.

Regarding CuZnSOD activity/expression relations, our results showed that CuZnSOD activity was positively correlated with its expression in patients with benign gynecological disorders (Figure 2A1), while in patients with hyperplasia and adenocarcinoma, a negative correlation was observed (Figure 2A2). This indicates that during the transition from benign to premalignant and malignant endometrial conditions, regulation of CuZnSOD activity also transits from expressional to dominantly post-translational level. 

Changes in CAT level during carcinogenesis, the mechanism of these changes, and their significance for course of the disease are still incompletely understood. The CAT activity and expression in a large number of tumors are found to be reduced in comparison to normal cells [32,33]. Our results are consistent with these findings, pointing to the decreased CAT level in both premalignant and malignant endometrial tissue (Figure 1B). Since increased level of H_2_O_2_ is associated with DNA damage, genetic instability [34,35], cell proliferation [36], and the regulation of apoptosis [37], a significant reduction of CAT recorded in endometrial hyperplasia may have an important role in cellular transition from premalignant to malignant state. 

Presumed mechanisms through which H_2_O_2_ affects the downstream paths of cellular signaling include modulation of interleukins [38], epidermal growth factor (EGF) [39], and tumor necrosis factor-α (TNF-α) [40], as well as inactivation of enzymes, such as protein tyrosine phosphatases (PTPs) [41], peroxidase [42], and catalase [43]. Beside playing an important physiological role in the stimulation of cell proliferation [44,45], H_2_O_2_ may also induce apoptosis associated with increased expression of p53, Puma, Noxa, and Bax in several cell types, including colon cancer and human cervical carcinoma HeLa cells [46,47]. It can also act as a promoter of neoplastic transformation through modulation of the PI3K/AKT signaling pathway due to oxidation of the PTP1B [48,49] and PTEN [50].

Although the exact mechanism of CAT regulation during the development of malignancy remains unknown, the absence of a positive correlation between its activity and level in all examined patients (Figure 2B1,B2) suggested that CAT activity in endometrial tissue is governed by a mechanism other than expression. Moreover, the results of our study indicated that this regulation is performed at the level of mRNA, whose amount (similar to protein level) was decreased in hyperplasia and adenocarcinoma groups (Figure 4B). 

It was shown that GPx plays an important role in cancer development, and that a loss of heterozygosity (LOH) at the GPx1 locus and changes in GPx expression may be the early events in cancerogenesis [51]. Epigenetic GPx alterations may also affect the process of malignant transformation. Previous studies showed that changes in GPx level could disrupt the balance between ROS production and AO defense, and thereby influence cancer development [52,53,54]. Our results also indicated that significant changes in GPx levels occurred during premalignant and malignant endometrial transformation. Specifically, the GPx protein level in patients with hyperplasia showed a tendency to increase compared to patients with polyps and myoma (Figure 1C). Similar alterations were observed at the level of mRNA (Figure 4C), indicating transcriptional regulation of this enzyme. Observed changes in GPx expression can affect the cell proliferation in several ways. It has been shown that GPx may inhibit apoptosis by removing hydroperoxides [55], or by alternating Bax/Bcl-2 ratio [56], which may occur as a relatively early event in tumor development [57].

In adenocarcinoma, the GPx protein level was not significantly changed compared to groups with benign uterine changes. However, a significant drop in Gpx protein level was recorded in the adenocarcinoma group compared to hyperplasias, although it was not accompanied by changes in its mRNA (Figure 1C and Figure 4C). A possible reason for this disproportion might be the increased proteasomal degradation, which occurs in conditions of mild oxidative stress, and the fact that protein sensitivity to proteolysis increases after exposure to oxidants. Similarly, expression of GPx was not increased in some other cancer types [58,59]. Considering the already increased H_2_O_2_ production in cancer cells [60], it may be concluded that simultaneous reduction of CAT and GPx in adenocarcinoma could additionally raise H_2_O_2_ level, and thus affect further progression and metastasis of cancer [57].

The positive correlation between GPx activity/level was detected in group of patients with polyps and myoma, but not in those with hyperplasia and adenocarcinoma (Figure 2C1,C2). This is in agreement with the findings that glutathione redox cycle is a major source of protection against low levels of oxidative stress (expected in benign endometrial changes), whereas CAT becomes more significant in protecting against severe oxidative stress (characteristic for premalignant and malignant cells) [61].

GR is considered as a central enzyme of cellular antioxidant defense, as it catalyzes the reduction of oxidized glutathione disulfide (GSSG) to the sulfhydryl form, glutathione (GSH). The GSH level is essential for preserving normal cell functions, preventing malignant transformation, and for resistance of already-transformed, malignant cells. Results of this study showed that compared to patients with polyps and myoma, levels of GR protein and GR mRNA were elevated in women with both types of hyperplasia and adenocarcinoma (Figure 1D and Figure 4D). The noticed increase might be significant for uterine carcinogenesis, as it is known that many types of malignant tumors are characterized by the increased expression of enzymes involved in the GSH metabolism [53]. Indirectly, the results of our study also pointed out the possible therapy resistance of uterine adenocarcinoma, as the high level of GSH was previously recorded in some radio- or chemo-resistant cancers, such as breast tumor, melanoma, and lung cancer [62,63,64]. Our previous data showed that GR activity also rises during premalignant and malignant processes in the human endometrium [11]. However, a positive association of the GR level and activity was observed only in patients with benign uterine changes (Figure 2D1). In groups with hyperplasias and adenocarcinoma, the tested GR activity was not correlated with GR protein level (Figure 2D2), and therefore was presumably not regulated solely by its expression. 

In general, according to our results presented in Figure 2 (A2, B2, C2, and D2), expression of AOE enzymes in hyperplastic and malignantly0transformed uterine tissue was not correlated with AOE activity, so increases in AOE protein levels do not necessarily lead to increases in their enzyme activities. Observed discordance could be the consequence of the oxidative stress, which although induces AOE expression by modulating different transcription factors [65], can also oxidatively alter the enzymes, thereby reducing or completely suppressing their activity [66,67]. As an outcome, oxidative stress presumed in premalignant and malignant tissue can cause accumulation of damaged, non-degradable proteins, without increasing their activity.

The LOOH is a well-known marker of oxidative stress [15,68] and peroxidative damage of membrane lipids. A part of our research concerning the relation of LOOH and AOE expression showed that only GPx and GR were positively correlated with LOOH level (Figure 3). This was not surprising, given that glutathione-associated metabolism is a major defense mechanism, not only against ROS, but also against their toxic products. Coordinated regulation of glutathione-dependent enzymes is also a common phenomenon and is achieved, at least in part, through the antioxidant responsive element (ARE), located in the promoters of many genes that are inducible by oxidative and chemical stress [69].

The results of our study have shown that in both groups with hyperplasia, the Nrf2 protein level was decreased in comparison to patients with benign and adenocarcinoma diagnoses (Figure 5B). Reduced Nrf2 level in the endometrium of patients with hyperplasia simplex and hyperplasia complex could be the cause of reduced detoxification and antioxidant capacities of these cells. Such a prooxidative environment certainly favors the formation of structural and functional cell damage, and formation of initial, premalignant lesion. Regarding the increase in Nrf2 level observed in the adenocarcinoma group, a similar increase has already been shown in the study of Chen et al. [70] in malignant tissue related to their precursor lesions. The authors have measured the Nrf2 expression in benign endometrium, endometrial cancers, as well as in their precursor lesions, and concluded that Nrf2 overexpression was closely associated with endometrial neoplasms with serous differentiation. Their study showed that alteration of Nrf2 expression may represent one of the early molecular events in endometrial carcinogenesis and that overexpression of Nrf2 may be used as a diagnostic marker in surgical pathology. Some other studies on human cancers have also shown that increase in the expression of Nrf2 can enhance the Nrf2-dependent mechanisms of protection, thereby intensifying the growth of cancer cells and providing them with chemoresistance to a large number of chemotherapeutic drugs [71,72].

Discrepancy between mRNA changes and the amount of its protein in adenocarcinoma tissue (Figure 5A,B) indicates the presence of an additional, posttranscriptional regulation of the Nrf2 level. The most likely mechanism is the interference in the correct binding of Keap1 to Nrf2, which prevents ubiquitination and proteasomal decomposition of Nrf2 molecules, a process that maintains Nrf2 at the physiological level. Possible causes of such a constitutive stabilization of Nrf2 molecules could be the mutations in the Nrf2 or Keap1 genes, already identified in many types of cancer [73,74], or the accumulation of proteins that interfere with Keap1-Nrf2 interactions, such as p62 and p21 [75,76]. However, the most likely cause is the methylation in the promoter region of Keap1 gene, as the connection between oxidative stress and hypermethylation of genes that can induce cancerogenesis was already shown in different cell types [77].

**Figure 6:**
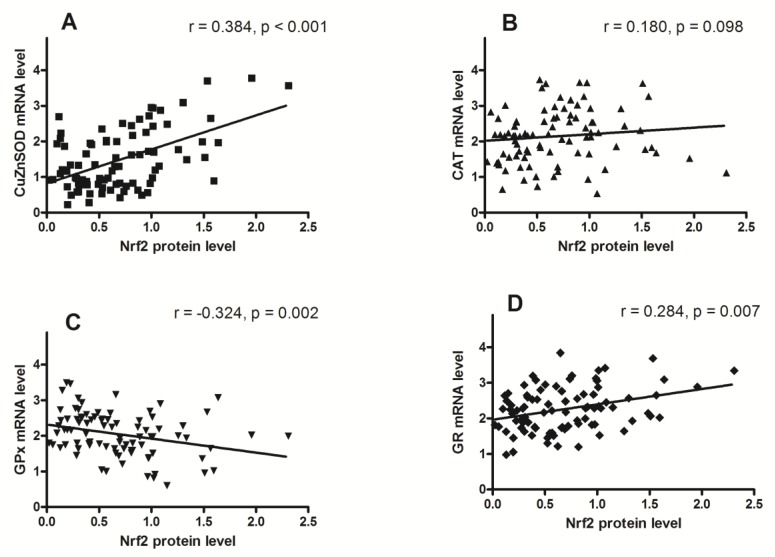
Scatter plots of Nrf2 protein level against mRNA levels of CuZnSOD (**A**), CAT (**B**), GPx (**C**), and GR (**D**). Protein levels were normalized to β-actin, and mRNA was normalized to POLR2A.

Results of our study showed positive correlation between the level of Nrf2 protein and mRNA levels of CuZnSOD and GR (Figure 6A,D). The highest level of Nrf2 observed in adenocarcinoma tissue is probably a key event in the induction of CuZnSOD and GR expression, since in that tissue the highest protein and mRNA levels for CuZnSOD and GR were also recorded. Contrary to those results, the protein level of Nrf2 was negatively correlated with GPx mRNA (Figure 6C), and it was not correlated with level of CAT mRNA (Figure 6B). It seems that some other mechanism or transcriptional factor dominates the Nrf2 transcriptional regulation of these two AOE. The induction effect of Nrf2 in these cases could be suppressed by competence, as it was experimentally shown on the mouse hepatocarcinoma cell line. In these cells, p53 binds to promoters containing ARE elements, and thus directly blocks the binding of Nrf2 and the transcriptional induction of several AOE genes [78]. Negative regulation of Nrf2 signaling was also described for some other molecules, such as NF-kappaB/p65 [79] and c-Myc [80].

To the best of our knowledge, this is the first study to investigate the expression of the four most important AOE enzymes and their regulation by the Nrf2 in various gynecological diseases. However, several limitations of our study should be noted. In addition to the relatively small number of samples, their distribution in groups was uneven. Also, due to unethical uterine sampling of healthy women, groups with hyperplasias and adenocarcinoma were compared with groups with benign changes, which can lead to false positive or false negative results in relation to healthy controls. Future studies should replicate the results with a larger sample set, and perhaps in an in vitro model system comparing benign and malignantly transformed cells with healthy ones.

## 5. Conclusions

The results of our study clearly showed that in the course of benign, premalignant, and malignant uterine transformation significant changes occurred in expression level of Nrf2, and consequently on transcriptional and translational levels of AOE. It is also evident that the impact of AOE expression on their enzyme activity depends not only on the type of the enzyme, but also on the type of endometrial transformation. Observed findings could contribute to a better insight into molecular mechanisms connecting oxidative stress with different gynecological disorders, and to a better understanding of therapeutic approaches based on altering the cellular redox status.

## Figures and Tables

**Table 1:** Patients characteristics.

Age (years)	25–34 (7)	35–44 (22)	45–54 (41)	55–64 (6)	65 ± 79 (3)
Parity (N)	0 (3)	1 (6)	2 (29)	3 (24)	>3 (17)
Abortions (N)	0(1)	1 (30)	2 (16)	3 (16)	>3 (16)
Uterine bleeding ^1^ (N)	None (26)	MP (19)	MR (15)	MPM (19)	
Diagnosis ^2^ (N)	PE (16)	UM (12)	SH (25)	CH (21)	ACE (5)

^1^ MP—Metrorrhagia prolongata, MR—Metrorrhagia recidivans, MPM—Metrorrhagia postmenopausi. ^2^ PE—polypus endometrii, UM—uterus myomatosus, SH—hyperplasia simplex endometrii, CH—hyperplasia complex endometrii, ACE—adenocarcinoma endometrii.
